# Intraventricular immune checkpoint inhibition with nivolumab in relapsed primary central nervous system lymphoma

**DOI:** 10.1093/noajnl/vdac051

**Published:** 2022-04-13

**Authors:** Leon D Kaulen, Christoph Gumbinger, Felix Hinz, Tobias Kessler, Frank Winkler, Martin Bendszus, Felix Sahm, Wolfgang Wick

**Affiliations:** 1 Department of Neurology, Heidelberg University Hospital, Heidelberg, Germany; 2 Clinical Cooperation Unit Neuro-Oncology, German Consortium for Translational Cancer Research (DKTK), German Cancer Research Center (DKFZ), Heidelberg, Germany; 3 Department of Neuropathology, Heidelberg University Hospital, Heidelberg, Germany; 4 Clinical Cooperation Unit Neuropathology, German Consortium for Translational Cancer Research (DKTK), German Cancer Research Center (DKFZ), Heidelberg, Germany; 5 Department of Neuroradiology, Heidelberg University Hospital, Heidelberg, Germany

**Sequencing studies have deciphered the genetic landscape of primary central nervous system lymphoma (PCNSL) paving novel therapeutic avenues in recent years.** **^1,2^ Frequent 9p24.1 copy number gains, the genetic locus of programmed cell death protein 1 (PD-1) ligands PD-L1 and PD-L2, were detected and suggestive of immune evasion *via* activation of PD-1 signaling.** **^1^ Systemic nivolumab, a monoclonal PD-1 antibody, was hence evaluated in a small retrospective PCNSL cohort yielding promising long-term responses.** **^3^ Intracranial efficacy of systemically administered monoclonal antibodies may however be impeded by an intact blood-brain barrier (BBB) resulting in lower cerebrospinal fluid (CSF) penetration. To bypass the BBB and reduce systemic toxicities, intraventricular/intrathecal immunochemotherapy is being investigated. Intrathecal nivolumab combined with its systemic administration was recently found safe and effective in melanoma with leptomeningeal dissemination,^4^ prompting us to evaluate intraventricular nivolumab for recurrent primary CNS lymphoma (PCNSL) in an elderly patient unable to tolerate aggressive systemic polychemotherapy. Intraventricular nivolumab achieved a lasting (>12 months) complete remission including parenchymal lesions distant from cerebrospinal fluid spaces. No toxicities or adverse events related to the mode of administration were noted. Our case suggests intraventricular nivolumab is active in recurrent parenchymal PCNSL. Together with detected 9p24.1 gains this argues for further prospective evaluation, for which our treatment protocol provides a framework.**

We describe a patient with unequivocal recurrence of a PCNSL who was subsequently treated with intraventricular nivolumab. Informed consent for placement of a Rickham reservoir, off-label use of nivolumab on a compassionate care basis, and for publication was obtained. Nivolumab (20 mg) was diluted in 3 ml of perseverative-free saline and administered following removal of equivalent CSF volumes. For induction, eight doses of nivolumab were injected at biweekly dosing intervals. For maintenance, it was administered every 4 weeks. The dose and intervals were selected based on clinical trial experiences in metastatic melanoma.^4^ Glucocorticoids were avoided before and during therapy. Staging magnetic resonance imaging (MRI) was performed after the first, fourth, and eighth dose during induction and every 3 months during maintenance therapy. International Primary CNS Lymphoma Collaborative Group criteria were used for radiological response assessment.^5^ Treatment-related toxicities were graded according to the Common Terminology Criteria for Adverse Events (CTCAE) version 5.

Treatment protocol, pathological, and radiological findings are summarized in Figure 1. A 77-year-old female patient with PCNSL was treated with first-line R-MP (rituximab, methotrexate, procarbazine) polychemotherapy in analogy of the PRIMAIN protocol.^6^ Therapy achieved complete radiographic response (CRR) but also caused grade III toxicities including leukopenia, infection, and diarrhea. The patient was then followed expectantly with scans every 3 months.

**Figure 1.**
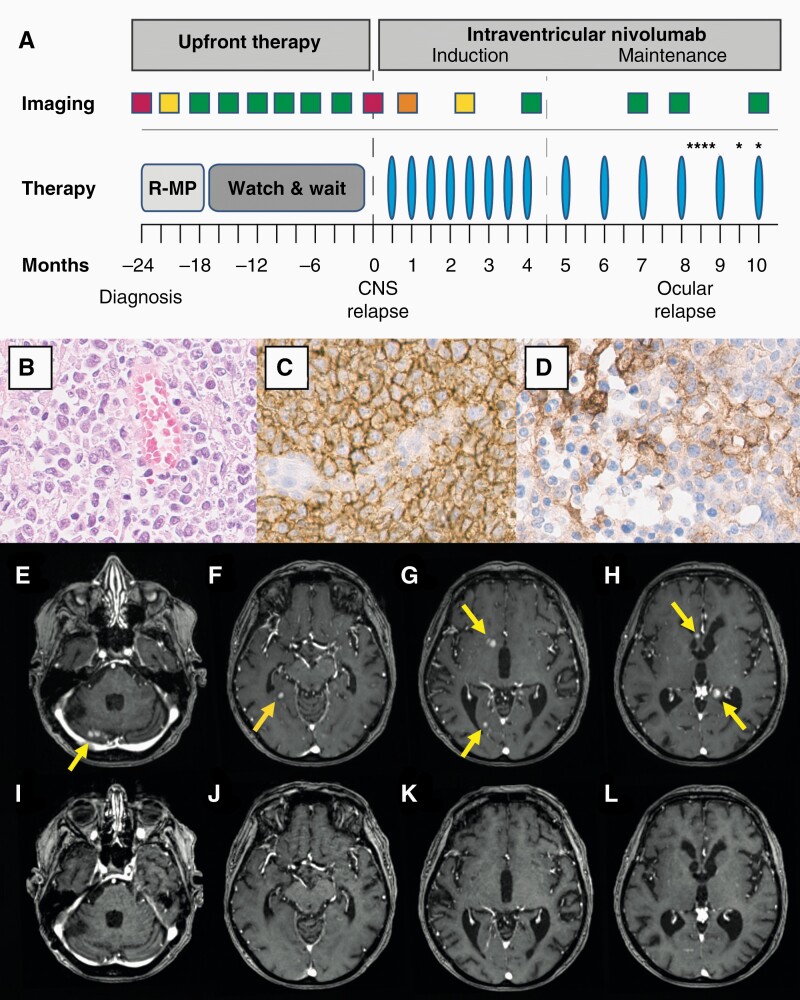
Treatment protocol, pathological, and radiological findings. (A.) Timeline and treatment protocol are shown. Rectangles indicate times of brain scans with colors representing imaging results (red: progressive disease; orange: stable disease; yellow: partial remission; green: complete remission). After rituximab, methotrexate, procarbazine (R-MP) polychemotherapy, the patient was followed expectantly. At multifocal parenchymal relapse, intraventricular nivolumab application (blue columns) was initiated with biweekly and 4-weekly dosing intervals for induction and maintenance treatment, respectively. Ocular disease was diagnosed after vitrectomy and treated with intravitreal methotrexate injections (asterisks). (B.) Hematoxylin and eosin staining of specimen obtained at initial diagnosis demonstrates a dense, pleomorphic lymphoid infiltrate with prominent nucleoli. Immunohistochemical staining indicates a predominant CD20^+^ differentiation (C.) with robust PD-L1 expression (D.). Original magnification ×400 (B.–D.). Contrast-enhanced T1-weighted images before intraventricular treatment (E.–H.) and following eight cycles (I –L.) of intraventricular nivolumab therapy are shown. Before PD-1 blockade (E.–H.), multiple homogeneously enhancing infra- and supratentorial lesions were present (yellow arrows). After eight cycles, MRI revealed complete response of multifocal parenchymal PCNSL (I.–L.).

Multifocal parenchymal PCNSL relapse was diagnosed 24 months after initial diagnosis when clinically silent, homogeneously enhancing lesions with restricted water diffusion were detected on MRI. CSF analysis revealed a normal cell count (3/mcf) and mildly elevated CSF/serum albumin ratio (12.9, normal <9.3) without evidence for malignancy on cytology and flow cytometry. Pathological re-evaluation of the specimen obtained at initial diagnosis revealed robust PD-L1 expression of lymphoma cells (Figure 1B–D). After Rickham reservoir placement, eight cycles of intraventricular nivolumab (20 mg) were administered with biweekly dosing intervals for induction therapy. MRI revealed stable disease, partial and complete remission following cycles 1, 4, and 8, respectively (Figure 1E–L). Of note, CRR included larger parenchymal lesions in proximity of the ventricles (<1 cm) but also lesions more distant from CSF spaces (<2-3 cm). Following eight cycles with biweekly intervals, nivolumab was administered every 4 weeks for maintenance therapy. Parenchymal disease remained in CRR in follow-up MRIs performed every 3 months. However, 8 months after initiation of nivolumab therapy reduced visual acuity of the left eye was noted. Ocular relapse was diagnosed following vitrectomy and treated with intravitreal methotrexate injections according to established protocols.^7^ Intraventricular nivolumab treatment was continued, and parenchymal lesions have remained in CRR for more than 12 months at the time of publication. Repeated CSF analyses revealed normalization of CSF/serum albumin ratio indicative of BBB recovery under intraventricular nivolumab treatment and stable cell counts without evidence for malignancy. No treatment-related toxicities or application-associated adverse events were noted.

We report successful intraventricular nivolumab therapy for recurrent multifocal PCNSL achieving long-term intracranial disease control without treatment- or application-related toxicities and adverse events. It is noteworthy that CRR included intraparenchymal lesions distant (2-3 cm) from CSF spaces. In line with previous studies assessing different agents, this observation continues to challenge the notion that intraventricular therapy is only active within their proximity and hence ineffective for intraparenchymal masses.^8^ This may suggest the involvement of the controversial glymphatic system in parenchymal drug delivery after intraventricular administration. As a result of arterial wall pulsatility, the glymphatic system is thought to transport CSF along with periarterial Virchow-Robin spaces deep into the parenchymal interstitium with drainage via perivenous spaces and meningeal lymphatic vessels to mediate CNS waste clearance.^9,10^ In a primate Alzheimer’s disease model, intraventricular β-secretase 1 (BACE1) antibody therapy achieved uniform intraparenchymal distribution, which was linked to the glymphatic system.^11^ Although its role in drug delivery particularly in disease states, which may affect CSF transport rates, warrants further investigation, it could similarly have allowed intraventricular nivolumab to sufficiently reach deep parenchymal lesions.

Like its systemic application, sole intraventricular administration appears effective in PCNSL.^3^ At a fraction of the overall dose, bypassing the BBB may allow higher CSF and lower systemic drug concentrations likely minimizing dose-dependent side effects outside the CNS. Reported CSF penetration following intravenous nivolumab ranged between 1:52 and 1:299.^12^ In comparison, a total of 360 mg nivolumab—less than a single intravenous infusion—were administered intraventricularly over the course of 12 months and 18 cycles of ongoing nivolumab treatment. Durable parenchymal response with isolated intraocular relapse, which responded well to local methotrexate treatment, could point to insufficient nivolumab delivery to the vitreoretinal compartment and/or development of resistance.

In agreement with this report, intrathecal nivolumab was found feasible and safe in 23 patients with metastatic melanoma. No application- and limited treatment-related toxicities (grade 3: 22%) without relevant neurological events were recorded.^4^ Notably, systemic polychemotherapy—particularly in the elderly—is often associated with severe hematological toxicities. The PRIMAIN study registered grade ≥3 toxicity in 81.3% and treatment-related death in 8.4% of participants.^6^ Similarly, three grade III toxicities were noted in our patient. Systemic nivolumab is associated with lower toxicity rates. However, in a trial evaluating intravenous PD-1 blockade for relapsed DLBCL (diffuse large B-cell lymphoma), treatment-related adverse events were nonetheless recorded in 62% with 24% experiencing grade ≥3 toxicities.^13^ Intraventricular immunochemotherapy could constitute a less hematotoxic alternative—particularly in the elderly and other cohorts who cannot tolerate aggressive systemic polychemotherapy.

Our study and recently detected 9p24.1 gains argue for further prospective evaluation in PCNSL, for which our treatment protocol may provide a framework. The efficacy for intraparenchymal lesions also makes it a candidate for other CNS malignancies with active PD-1 signaling.

## Data Availability

Data are available from the corresponding author upon request by qualified researchers.
